# From Diversity to Coordination: A European Approach to COVID-19

**DOI:** 10.1017/err.2020.36

**Published:** 2020-04-16

**Authors:** Alessio M. PACCES, Maria WEIMER

**Affiliations:** * Amsterdam Law School and Amsterdam Business School, Amsterdam Center for Law and Economics, University of Amsterdam, Amsterdam, The Netherlands; email: a.m.pacces@uva.nl.; ** Amsterdam Law School, Amsterdam Centre for European Law and Governance, University of Amsterdam, Amsterdam, The Netherlands; email: m.weimer@uva.nl.

## Abstract

The COVID-19 pandemic is changing the face of Europe. Member States’ divergent responses to this crisis reveal a lack of unity in the face of a humanitarian catastrophe. At best, this undermines the effectiveness of health protection within the European Union (EU). At worst, it risks breaking up the Union altogether. Divergent national responses to COVID-19 reflect different national preferences and political legitimacy, and thus cannot be completely avoided. In this article, we argue that these responses should be better coordinated. Without coordination, the price for diversity is high. Firstly, there are damaging spill-overs between Member States, which undermine key pillars of European integration such as the free movement of persons and of goods. Secondly, national policy-making is easily captured by local interest groups. Our proposal is that the EU indicates – not mandates – a European exit strategy from asymmetric containment policies of COVID-19. In particular, the EU should help Member States procure and validate tests for infection and immunity. The EU should also indicate ways in which testing could be used to create safe spaces to work, thereby restoring the free movement of persons and of goods. We see a great advantage in such EU guidance: it could improve mutual learning between Member States, which have faced different timings of the epidemic and learned different lessons. Although the local political economy has so far delayed learning and undermined cooperation, the EU can mitigate both effects and indicate the way for Europe to resurrect united from the ashes of COVID-19.

The COVID-19 pandemic is rapidly changing the face of Europe. In a European Union (EU) founded on the idea of free movement, borders are being erected. In an interconnected world, countries are trying to shield themselves from a threat that is global and does not respect border checks nor carry a passport. The COVID-19 pandemic is a transboundary challenge of proportions unseen since the creation of the EU. Arguably, challenges like these are why we have the EU in the first place: to enable effective collective action in the face of transboundary problems that no Member States can address on their own. The EU is a global regulatory power, especially in the field of risk regulation, and has an institutional structure in place to address public health emergencies. However, paradoxically, in the current situation national governments are calling the shots. Their divergent responses to this crisis seem to reveal a lack of unity in the face of a humanitarian catastrophe. At worst, they risk breaking up the EU altogether.

What explains the diversity of national public health responses to COVID-19,^1^ and what is the price Europe is paying for it? Moreover, what is the appropriate role of the EU in this crisis? In this essay, we will address these questions by combining two perspectives on the pandemic: a risk regulation and an economic perspective. Our analysis reveals certain uncomfortable truths about the interplay between science and politics on the one hand and the need to balance European unity with pervasive diversity when it comes to national attitudes to risk on the other. The main argument is this: there are good reasons for why Member States are in charge of public health responses to pandemics and other health threats. The EU does not have legal nor a sufficiently strong democratic-political authority to take the lead on COVID-19, especially given the current scientific uncertainty. However, decentralised and uncoordinated crisis management causes significant spill-overs that are damaging to EU public health, the economy and to core European values. The EU must turn diversity into a strength and do what it does best: enable coordination and mutual learning and organise solidarity. After initial mistakes and weak coordination in the first weeks of the crisis, the EU must now take the lead in orchestrating a coordinated and safe European exit strategy with a focus on testing. The EU offers the necessary tools to accomplish that. Member States must use them.

## I. Coping with uncertainty and the politics of COVID-19: lessons from EU risk regulation

Containing the spread of COVID-19 is an exercise of emergency risk regulation on an unprecedented scale. Under normal circumstances, EU risk regulation is a challenging task.^2^ The current crisis takes these challenges to a new level. Before taking measures, policy-makers have to understand the nature and magnitude of a given risk, for which they rely on scientific advice. Science is also required to identify a risk (ie the likelihood of harm) in the first place. COVID-19, for example, is an “invisible risk”,^3^ because only special tests can identify who is infected, and our lack of understanding of the real numbers of infections is an enormous challenge. While science is crucial, it is often uncertain and inconclusive. There is substantial uncertainty with regards to the epidemiological characteristics of COVID-19 (eg how the disease spreads, whom it affects, death rate, etc.).^4^ As a consequence, policy-makers must make decisions in the face of incomplete and rapidly changing scientific knowledge. This exacerbates the political challenge of risk regulation, as is painfully illustrated by COVID-19, as containment policies entail enormous economic, social and human costs. Policy-makers must weigh trade-offs and make political choices about how these costs ought to be distributed. This produces conflicts and tests European solidarity, as we will discuss further below.

The EU has experience with navigating such challenges. EU law provides a number of principles that aim to address them. Public health responses to COVID-19 have to follow the principle of risk analysis.^5^ This principle ensures a balance between science and politics by dividing decision-making into two stages. First, a *risk assessment* – carried out by scientific experts – ensures the scientific objectivity and quality of regulation.^6^ Second, *risk management* – a task assigned to policy-makers – ensures the political responsibility and democratic legitimacy of regulation. While scientific advice is crucial, it remains advisory in nature. Risk managers can deviate from it with good reasons and must take a range of non-scientific considerations into account. To paraphrase a famous pronouncement of the EU general court in *Pfizer,* the risk managers’ public authority derives from their democratic legitimation (ie control by and accountability towards representative democratic institutions). Scientific risk assessors, “although they have scientific legitimacy, have neither democratic legitimacy nor political responsibility. Scientific legitimacy is not a sufficient basis for the exercise of public authority”.^7^ This does not diminish the importance of scientific advice, but recognises a core dilemma: science, while performing crucial cognitive tasks, cannot provide answers to political (and hence normative) questions.^8^ Risk managers carry the political responsibility of deciding whether action is warranted and what kind of action. This entails difficult political, economic and ethical choices, which vary from one society to another. Which risks societies are willing to accept and what the levels of protection should be are decisions for democratically accountable institutions.^9^

Risk managers are, however, not fully free to determine how much health protection is desirable. Both the EU and Member States, when managing COVID-19 risks, must ensure a high level of public health protection.^10^ They must also respect the precautionary principle, which is triggered by the scientific uncertainty around COVID-19. The principle requires early and anticipatory action. While a zero-risk approach is not allowed, the precautionary principle requires policy-makers to protect public health without having to wait until the reality and seriousness of risks become fully apparent or until the adverse effects materialise.^11^ It also requires them to prioritise public health over economic interests.^12^ With pandemics, the need for early action even before cases start to go up is extremely important,^13^ even if they seem like an overreaction at first. While precautionary measures must be proportionate, it is questionable how much room for proper proportionality there is in emergency situations.^14^ To paraphrase a World Health Organization (WHO) expert, waiting for perfection when containing a pandemic means being too late.^15^

## II. The EU division of tasks for public health emergencies

EU law of public health emergencies assigns the tasks of risk assessment and risk management to different levels of government. While the EU ensures a more or less harmonised risk assessment of COVID-19, national governments are primarily responsible for managing the pandemic, including for the application of the precautionary principle. This has to do with EU competences. It is well known – yet worth stressing – that the EU is a Union of conferred powers. According to the EU Treaties, health policy is a national competence.^16^ Member States as the signatories of the Treaties have granted the EU only limited powers for public health emergencies. Under the current EU regulatory framework,^17^ the role of the EU is to support national crisis management. It mainly acts as a hub for rapid information exchange and coordination of national crisis responses.

There are, in principle, good reasons for this division of tasks between the EU and the Member States. The organisation of national health systems is complex and varies across Member States. Different approaches are rooted in national culture and history. In federal Member States, such as Germany, competences for public health are a matter of sub-federal entities (eg the Länder). National governments are best placed to assess the availability of resources and critical infrastructures, training of staff and equipment, as well as the expected behaviour of citizens in a crisis. After all, trust in doctors and governments is shaped by cultural attitudes and is a factor in determining the effectiveness of emergency measures, such as social distancing and lockdowns. It is therefore not surprising that Member States adopt different responses to COVID-19.^18^ They carry the political responsibility for their crisis management vis-à-vis their citizens. EU institutions, especially the European Commission, do not possess the same level of political authority,^19^ nor are they subjected to the same kind of democratic accountability as national governments.

The lack of proper EU risk management powers in health emergencies could be seen as a weakness. In contrast, we hold that, under certain conditions, this could become a source of strength. If used effectively by the Member States, EU mechanisms of coordination and support *can* foster very powerful mutual learning and solidarity while respecting legitimate diversity.^20^ How well this potential is realised depends on the political will of national governments. Divergent national responses to COVID-19 cannot be avoided. Yet, they can and must be better coordinated. Without effective coordination, there is a high price to pay for diversity, an issue to which we turn next.

## III. The price of diversity: an economic perspective

According to the economics of federalism, which level of government should decide on COVID-19 depends on the tension between different criteria.^21^ For instance, diverging local preferences support decentralised solutions, but may result in cross-border spill-overs. Different responses to the epidemic may allow countries to learn from each other, but also result in conflicts of interest that undermine cooperation. In this section, we discuss the drawbacks of decentralised policy-making on COVID-19.

Because different communities attach different values to health protection and face different costs from containment measures, decentralised risk management meets the preferences of a society more efficiently. Even under uncertainty,^22^ the understanding of the precautionary principle varies considerably across countries. For example, at the time of writing,^23^ The Netherlands and Sweden have laxer containment policies than the rest of the EU, admittedly reflecting a lower risk and uncertainty aversion than other countries. This variation was higher in the beginning of the outbreak.

The disadvantage of decentralised containment policies is cross-border spill-overs. For instance, if a Dutch resident can freely travel to Belgium, having stricter containment measures, Belgian residents bear the effects of Dutch risk management.^24^ The response to this problem has been to reintroduce border controls within the EU, with the exception of countries having comparable containment policies. This admittedly temporary measure may not seem harmful when most people in the world are encouraged to stay at home. However, border checks and restrictions are here to stay for some time because when measures are lifted in some countries they will still be in place in some others, and this dissonance might be repeated in several rounds as part of a gradual exit strategy until a vaccine is found. The significant restriction of the free movement of persons is likely to undermine everyone’s sense of belonging to the EU. Moreover, closing borders reduces interstate labour mobility, which is part of the bigger problem of COVID-19 as a global supply shock.

A second cross-border spill-over of different risk management choices stems from the disruption of supply chains.^25^ COVID-19 is not only a medical but also an economic emergency. Compared to a “normal” recession, output will fall also because some production must be halted or reduced to prevent excessive contagion. Inputs, such as equipment and labour incompatible with social distancing, get scarcer and reduce the supply of certain goods.^26^ This already affects countries differently, depending on their specialisation and openness to trade, but the difference will be exacerbated by the different timing of containment and exit policies. Countries in lockdown must cope with shortages of goods and impose them on others not only because of underproduction, but also due to hoarding, export bans and other restrictions. Reduced mobility of scarce inputs is not only inefficient from a global perspective, but also incompatible with the EU internal market. It undermines the free movement of goods. Such freedom has already been restricted with respect to medical equipment, prompting a response by EU institutions.^27^ We expect an increase of these protectionist reactions as more goods become scarce with the spreading of the disease. This reflects a more general problem: asymmetric COVID-19 policies create conflicting interests that, in turn, undermine cooperation between states.

## IV. Why regulatory competition does not work: the political economy of delay

Diversity in approach and timing of policies against COVID-19 could lead countries to compete with each other and learn the optimal reaction (also considering local preferences) faster than if they were to coordinate. Given the uncertainty around COVID-19, the benefit of a spontaneous convergence towards optimal solutions would outweigh the drawbacks of cross-border spill-overs and conflicts of interest because these would also quickly disappear.^28^

We doubt, however, that effective solutions would emerge from regulatory competition soon enough. Firstly, scientific information is produced too slowly compared to the speed of the outbreak. Secondly, policy-makers react to the saliency of the outbreak rather than to scientific information. As a result, the optimal amount of convergence occurs with a delay, if at all, leaving countries to deal with unnecessary conflicts of interest and spill-overs between them.

Because COVID-19 spreads so rapidly, scientists lag behind with the generation of relevant information about the virus. Data on contagion, hospitalisation and deaths come in imprecisely and with delay. Nevertheless, a team of epidemiologists at Imperial College London (ICL) has recently modelled contagion and the impact of policies thereupon. In the absence of widespread testing or a vaccine, there are two policy options for containment: mitigation, which seeks to slow down contagion by moderate social distancing to build herd immunity; and suppression, which seeks to avoid contagion as much as possible, including lockdowns, until a vaccine is found. One ICL study found that, in the USA and the UK, mitigation policies would still lead to a sheer number of secondary deaths as the healthcare system becomes congested: people who would otherwise survive die lacking access to intensive care units (ICUs).^29^ The lesson for countries having a similar ICU surge capacity is that suppression policies are the only way to avoid congestion in the initial phase of contagion. Another finding from ICL is that the suppression policies implemented in Italy averted up to 80,000 deaths in a month.^30^ There is a method in economics to calculate how much a life is “worth” for a specific society: the value of statistical life (VSL).^31^ However coarse and controversial it may be, particularly because it consistently *under*estimates the value of human life for lower-income people,^32^ the VSL gives an idea about the economic benefit of averted deaths. In Italy, where the VSL is approximately US$5 million and the GDP about US$2 trillion, one month of lockdown was worth up to 20% of GDP.

Confronted with COVID-19, politicians worldwide have initially argued that there is a trade-off between protecting health and protecting the economy.^33^ This sounds odd in light of the above back-of-the-envelope calculations. Even assuming that the epidemic does not change the behaviour of individuals, the life-saving benefit of containment policies largely exceeds foregone output, at least insituations comparable to Italy. Moreover, the above-mentioned trade-off reflects a misconception. It wrongly assumes that people adopt social distancing only if the government tells them to do so.^34^ This reasoning neglects that, facing widespread contagion along with the likely congestion of the healthcare system, many individuals would implement social distancing anyway and fail to produce output because they get sick, die or are afraid of either. Compared to government intervention, individual reaction and panic would have a much worse impact on the economy – not to mention issues of inequality and social unrest. Even in the absence of precise estimates of contagion and mortality rates, there is no economic reason to delay containment. Moreover, delays are also contrary to the precautionary principle.^35^ However, policy-makers invariably delayed action until congestion of the healthcare system became a foreseeable problem in their own country. Policy-makers have not learned from the experience of other countries.

Political economy explains these delays.^36^ Politicians tend to react to an emergency rather than anticipating it. This is particularly the case with non-linear phenomena, such as epidemics. Contagion starts very slowly, but becomes politically salient only when it escalates. People would consider unwarranted any restriction introduced before the spill-overs from contagion become visible. In that phase, interest groups bemoaning the economic cost of containment prevail in the political discourse.^37^ This undermines policy-makers’ learning from scientists and from the experience of other countries. Denial is only reversed when the gravity of the situation becomes salient: the prospect of letting citizens die because of ICU congestion is not politically palatable. But then the disease spreads exponentially, and there is no time to ponder the policy options.

As convergence has not happened quickly enough, coordination would seem preferable to regulatory competition. Unfortunately, delays have undermined the incentive of countries to cooperate. Countries may be pursuing mitigation or suppression of contagion. These strategies are incompatible and, for the time being, unstable. Mitigation without massive testing may have to turn into suppression when ICU capacity nears saturation, as happened in the UK. Suppression is not sustainable, both psychologically and economically, for as long as it seems likely to take until a vaccine is found.^38^ Countries that have suppression in place, such as Italy, will have to suspend it and reinstate it depending on ICU inflows. Therefore, countries facing COVID-19 have different interests at different times, exacerbating spill-overs and potential conflicts. Yet, because containment policies are unstable, every country needs an exit strategy. Coordinating on the exit strategy would remedy spill-overs and conflicts while improving the effectiveness of the response to COVID-19. In the next section, we argue that the EU could play a decisive role in such coordination.

## V. Making the most out of Europe

The EU offers important tools and institutional structures for coordination, mutual learning and solidarity. Given these tools, at no other time in history could the Member States be better prepared, in principle, to face a global pandemic such as COVID-19. Yet they were not. The success of the European fight against COVID-19 – and probably the survival of the European project as such – depends on Member States making the most out of Europe under the current circumstances.

Let us first look at the tools that the EU makes available. Drawing lessons from previous outbreaks, such as SARS and bird flu (H1N1), the EU has established a legal framework for public health emergencies: the Cross-Border Health Threats Decision.^39^ This aims to contribute to a high level of public health protection in the EU by ensuring the coordination of both risk assessment and risk management in public health emergencies. An EU agency, the European Centre for Disease Prevention and Control (ECDC), ensures the coordination of scientific advice between the EU and national risk assessors. It monitors the disease outbreak, produces continuous risk assessments for the EU population as a whole and provides guidance for risk management. It is also an important source of comparable epidemiological data for the Member States – an aspect crucial for mutual learning about what may or may not work in the fight against the virus.^40^

Moreover, the Commission has created a new expert group, the EU advisory panel on COVID-19.^41^ The panel directly advises Commission president Ursula von der Leyen on measures to be taken at the EU level. It also helps (in coordination with the ECDC) the Commission develop guidance for Member States to ensure consistent, science-based and coordinated national risk management. Since the panel took up work on 17 March 2020, the Commission has published the first EU-wide recommendations for community measures, testing strategies and health systems resilience.^42^

In terms of risk management, Member States are obliged to coordinate their COVID-19 measures in the so-called EU Health Security Committee, composed of national health ministers and chaired by the Commission.^43^ The Health Security Committee is a crucial forum for mutual consultation and regular information exchange among Member States. Ideally, scientific advice (from the ECDC and the COVID-19 panel) and national risk management should be linked here with national health ministries explaining how they have considered EU advice as well as the reasons for their choices of containment policies. This should also allow for some degree of external accountability for spill-overs:^44^ Member States being made aware and considering the effects of national policies on other Member States. Moreover, Member States should use the Health Security Committee as a site to report both successes and failures of national approaches to COVID-19 and be willing to adjust their policies accordingly. This is where diversity can turn into a strength. Sharing national experiences in the fight against the pandemic in the Health Security Committee may improve mutual learning,^45^ which is key in the fight against COVID-19.

The Committee is also crucial for informing the Commission about national healthcare capacities and needs, thereby triggering mechanisms of EU-wide public health solidarity. The EU Health Threats Decision foresees such mechanisms via the possibility of voluntary public procurement of medicinal countermeasures and medical equipment. Preventing harmful competition for vaccines and medical equipment between EU member states is crucial and addresses the spill-overs resulting from the disruption of the free movement of goods and of supply chains.^46^ A joint EU procurement of these goods gives Member States a strong bargaining position towards manufacturers. It also allows for the distribution of such goods to places where they are most needed across Europe. Currently, the EU has launched four joint procurements of personal protective equipment (eg masks, ventilators and testing kits).^47^ Because EU law only foresees voluntary public procurement, only 25 Member States are participating in these initiatives. The voluntary participation is a problem, as it undermines the EU’s capacity to act quickly and in the common European interest. Calls for more EU powers to purchase, store and allocate medicines and equipment to fight infectious diseases are therefore justified.^48^ They are not in conflict with national responsibilities for risk management, but rather are in support of them.

There are other ways in which the EU can address the spill-overs of decentralised risk management of COVID-19 while at the same time strengthening the idea of Europe as a “*Schicksalsgemeinschaft*”.^49^ Defining common criteria for legitimate border restrictions is key, as the Commission has done.^50^ The EU-wide scheme for export authorisation for medical equipment has helped with lifting national export bans initially imposed by some Member States. The creation of so-called “green lanes”^51^ to ensure the free flow of critical goods and personnel contributes to public health protection and helps overcome supply problems in the EU. This shows that the EU can be very creative with how it uses its powers and can also protect public health by using competences in other fields of policy, above all the internal market.

**Figure 1.**
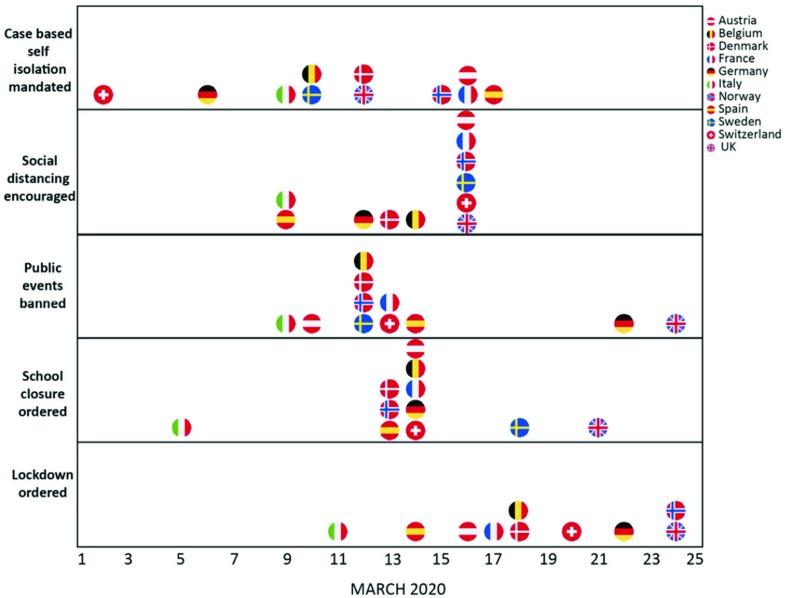
Timing of government interventions in 11 European countries (source: Imperial College London).^55^

Things are moving quickly, and it is difficult to assess how well coordination in the Health Security Committee actually works at present.^52^ Judging by the initial cacophony of “us first” responses, until approximately mid-March,^53^ there was little coordination. Things do seem to have improved, however. National containment measures and approaches still differ, but they are converging, with most Member States having adopted a combination of social distancing, school closures, public events bans and (more or less stringent) lockdowns.^54^ This is partially due to timing: the more the virus spreads across the EU, the more Member States move to stringent measures (Figure 1).

Yet this also signals improved cooperation at the EU level. The COVID-19 advisory panel seems to have given scientific guidance more weight across the EU. The Commission recommendations drafted with its input are discussed in the Health Security Committee, and Member States are asked to provide feedback and to report on how they have implemented the guidance. The Commission also asks Member States to identify priorities for the future work of the panel, thus steering its scientific work towards more responsiveness to local needs, which is likely to improve the uptake of its advice in the future. The Committee also coordinates the redistribution of patients and health professionals across the EU in order to address more acute needs in some regions/countries.^56^ There are positive signs that, although delayed, the work of the Health Security Committee is going in the right direction.

Going forwards, all efforts should be aimed at designing and implementing a European exit strategy on how to lift social distancing measures in a safe way. Testing the wider population to determine contagion and immunity is key to any such exit strategy.^57^ On the one hand, creating a safe space to work, in which people are either immune or not infected (and distanced from anyone who could be), is crucial to restarting the economies after the contagion is under control, without risking a second peak of the outbreak. On the other hand, having an EU approach to labour mobility and physical production could overcome the current and foreseeable restrictions to the free movement of persons and goods. For instance, workers from different Member States could participate in EU “green zones” that are free of contagion and so produce scarce goods.^58^ Solutions of this kind would complement national approaches to the exit strategy, but also promote more solidarity between Member States. Although we have not addressed this, economic solidarity between EU Member States is in fact a precondition to restarting the European economies by way of exit from the containment measures.^59^

After initial mistakes and weak cooperation with regards to putting containment measures in place, it is now high time, and not less crucial, to agree on a coordinated European approach to the exit strategy. The Commission and the Health Security Committee will have to play an important role in this regard.^60^ The EU must help the Member States obtain the equipment for extensive testing (ie assessing EU-wide needs and availability of testing, organising joint procurement of testing kits, negotiating with industry and funding acquisition). Moreover, test validation and the criteria for relaxing the containment measures must be coordinated between Member States in order for the exit strategies to avoid – and even remedy – the negative spill-overs of the past.

As this paper went to press, the Commission published a roadmap for a coordinated EU exit strategy.^61^ Member States initially resisted the publication of this document.^62^ Some, such as Austria and Denmark, announced unilateral plans to relax COVID-19 lockdowns that differed from the plans of other EU countries.^63^ The insistence of the Commission addresses our key concern: the longer the EU waits, the less room will there be for coordination. This could result in long-lasting damage to free movement and EU solidarity. Public health protection is equally at risk if national lockdowns are relaxed too quickly and are based on political opportunism or economic lobbying rather than commonly agreed science-based criteria. Differently from the former, the latter take the still pervasive scientific uncertainty surrounding the virus into account.^64^ The initiative of the Commission addresses these concerns. It is a commendable first step towards delineating a common European framework on how to gradually lift confinement measures based on common principles of scientific advice, coordination and solidarity. At the same time, however, the roadmap still leaves ample margins for Member States to continue restrictions of free movement in the presence of asymmetric situations and policies.^65^ More EU leadership seems to be in order, particularly in the design and mutual recognition of testing and tracing policies.^66^ Ultimately, the ball is now in the Member States’ court. They must act in the common European interest and follow the Commission’s lead.

## VI. Conclusions

The fight against COVID-19 is a marathon, not a sprint. Europe’s survival will depend on how it handles the exit from this crisis. In this article, we noted the challenge stemming from different health policies in the EU. While this difference reflects national preferences and political legitimacy, it has produced negative spill-overs between Member States. We propose to turn this challenge into an opportunity.

Our proposal is that EU institutions indicate – not mandate – a European exit strategy from asymmetric, albeit gradually converging, containment policies of COVID-19. In particular, the EU should help Member States procure and validate tests for infection and immunity. The EU should also indicate ways in which testing could be used to create safe spaces to work, thereby restoring the free movement of persons and of goods. We see a great advantage in such EU guidance: it could improve mutual learning between Member States that have faced different timings of the epidemic and learned different lessons. Although, as we showed, the local political economy has so far delayed learning and undermined cooperation, the EU can mitigate both effects and indicate the way for Europe to resurrect united from the ashes of COVID-19.

## Footnotes

1 In this article, we do not discuss other responses, such as the policies addressing the socioeconomic and the financial consequences of the crisis.

2 See M Weimer , Risk Regulation in the Internal Market (Oxford, Oxford University Press 2019 ).

3 See M Weimer , “The Origins of ‘Risk’ as an Idea and the Future of Risk Regulation” (2017) 8(2) European Journal of Risk Regulation 10 .

4 See European Centre for Disease Prevention and Control, seventh risk assessment, p 19 <https://www.ecdc.europa.eu/sites/default/files/documents/RRA-seventh-update-Outbreak-of-coronavirus-disease-COVID-19.pdf>.

5 See A Alemanno , “Regulating the European Risk Society” in A Alemanno et al (eds), Better Business Regulation in a Risk Society (Berlin, Springer 2013) pp 37–56 .

6 Pfizer, Case T-13/99 <http://curia.europa.eu/juris/liste.jsf?language=en&num=t-13/99>.

7
ibid, para 201.

8 See C Joerges , “Science and the Management of Risks to Health at the National, European, and International level” (2001) 7 Columbia Journal of European Law 1 .

9 Bayer, Case T-429/13 <http://curia.europa.eu/juris/liste.jsf?num=T-429/13&language=EN> and T-451/13 <http://curia.europa.eu/juris/liste.jsf?num=T-451/13&language=EN>.

10 See Arts 9 and 168 TFEU.

11 BSE, Case C-157/96 <https://eur-lex.europa.eu/legal-content/HR/TXT/?uri=CELEX:61996CJ0157> and C-180/96 <https://eur-lex.europa.eu/legal-content/HR/TXT/?uri=CELEX:61996CJ0180>.

12 Bayer, supra, note 9.

13 See G Pisano, R Sadun and M Zanini, “Lessons from Italy’s Response to Coronavirus”, *Harvard Business Review*, March 2020 <https://hbr.org/2020/03/lessons-from-italys-response-to-coronavirus>.

14 Although cost–benefit analysis has also shown by now the appropriateness of early and stringent containment measures, see M Greenstone and V Nigam, “Does Social Distancing Matter?” University of Chicago, Becker Friedman Institute for Economics Working Paper No. 2020-26. See also Section IV below.

15 Dr Michael J Ryan at WHO press conference, 13 March 2020 <https://www.youtube.com/watch?v=AqRHH6e-y6I>.

16 Arts 2 (5), 6 a) and 168 TFEU.

17 Decision No 1082/2013/EU of the European Parliament and of the Council of 22 October 2013 on serious cross-border threats to health (hereinafter the “Health Threats Decision”).

18 See <https://www.theguardian.com/world/2020/mar/12/how-do-coronavirus-containment-measures-vary-across-europe?CMP=Share_iOSApp_Other>.

19 Weimer, supra, note 2.

20 On the diversity challenge in EU risk regulation, see ibid.

21 See generally R Van den Bergh , “Farewell Utopia? Why the European Union Should Take the Economics of Federalism Seriously” (2016) 23(6) Maastricht Journal of European and Comparative Law 937 .

22 On the difference between risk (which can be calculated) and uncertainty (which cannot), see F Knight , Risk, Uncertainty and Profit (1921 – Reprints of Economic Classics, New York, 1964 ).

23 Beginning of April 2020.

24 “Belgian Town Turns Coronavirus Anger on the Dutch’, *Politico*, 24 March 2020 <https://www.politico.eu/article/belgium-is-tired-of-dutch-coronavirus-tourists>.

25 See, eg, “Coronavirus and Supply Chain Disruption: What Firms Can Learn”, *Knowledge@Wharton*, 17 March 2020 <https://knowledge.wharton.upenn.edu/article/veeraraghavan-supply-chain>.

26 R Baldwin, “The Supply Side Matters: Guns versus Butter, COVID-style”, *Vox.EU*, 22 March 2020 <https://voxeu.org/article/supply-side-matters-guns-versus-butter-covid-style>.

27 See Section V below.

28 This has been powerfully argued by H Eidenmüller, “The Race to Fight COVID-19: On the Desirability of Regulatory Competition”, *Oxford Business Law Blog*, 31 March 2020 <https://www.law.ox.ac.uk/business-law-blog/blog/2020/03/race-fight-covid-19-desirability-regulatory-competition>.

29 N Ferguson, D Laydon and G Nedjati-Gilani. “Report 9 – Impact of Non-Pharmaceutical Interventions (NPIs) to Reduce COVID-19 Mortality and Healthcare Demand”, Imperial College London preprint (16 March 2020).

30 S Flaxman, S Mishra and A Gandy. “Report 13 – Estimating the Number of Infections and the Impact of Non-Pharmaceutical Interventions on COVID-19 in 11 European Countries”, Imperial College London preprint (30 March 2020).

31

WK Viscusi and CJ Masterman , “Income Elasticities and Global Values of a Statistical Life” (2017) 8(2) Journal of Benefit–Cost Analysis 226 .

32

A Arcuri , “Risk Regulation” in AM Pacces and RJ Van den Bergh (eds), Encyclopedia of Law and Economics (Cheltenham, Edward Elgar 2012) pp 321–22.

33 This is still part of the policy debate; see “A Grim Calculus – Covid-19 Presents Stark Choices between Life, Death and the Economy”, *The Economist*, 2 April 2020 <https://www.economist.com/leaders/2020/04/02/covid-19-presents-stark-choices-between-life-death-and-the-economy>.

34 W Knight, “A Rush Back to ‘Normal’ Would Be the Blunder of the Century”, Interview to Larry Summers, *Wired*, 28 March 2020 <https://www.wired.com/story/rush-back-to-normal-blunder-of-the-century>.

35 See Section II above.

36 See, eg, L Zingales, “Captured Western Governments Are Failing the Coronavirus Test”, *Pro-Market*, 13 March 2020 <https://promarket.org/captured-western-governments-are-failing-the-coronavirus-test>.

37 “The Swedish Exception: Why Swedes Are Not Yet Locked Down”, *The Economist*, 4 April 2020 <https://www.economist.com/europe/2020/04/04/why-swedes-are-not-yet-locked-down>.

38 A minimum of 18 months seems necessary for a COVID-19 vaccine to be ready for distribution; see, eg, “WHO Expert: Finding and Distributing COVID-19 Vaccine in 18 Months Would Be ‘historic’”, *Euractiv*, 20 March 2020 <https://www.euractiv.com/section/coronavirus/interview/who-expert-finding-and-distributing-covid-19-vaccine-in-18-months-would-be-historic>.

39 See above n. 17.

40 See G. Pisano, R. Sadun and M. Zanini, ‘Lessons’ above n. 13.

41 <https://ec.europa.eu/transparency/regexpert/index.cfm?do=groupDetail.groupDetailDoc&id=39740&no=1>.

42 <https://ec.europa.eu/info/live-work-travel-eu/health/coronavirus-response/public-health_en#a-european-team-of-covid-19-experts>.

43 Art. 11 of the Decision, see above n 17.

44 On this concept, albeit in the context of the WTO, see M. Weimer , ‘Reconciling regulatory space with external accountability through WTO adjudication – trade, environment and development’, Leiden Journal of International Law 30(4) (2017) 901–924 .

45 See G. Pisano, R. Sadun and M. Zanini, ‘Lessons’ above n. 13.

46 See Section III above.

47 It has also used an EU civil protection mechanism, rescEU, to set up a stockpile of medical equipment; see <https://ec.europa.eu/commission/presscorner/detail/en/ip_20_476>.

48 A de Ruijter et al, “Give the EU More Power to Fight Epidemics”, *Politico*, 26 March 2020 <https://www.politico.eu/article/coronavirus-eu-power-pandemic>.

49 A community of fate.

50 See Commission Guidelines for border management measures to protect health and ensure the availability of goods and essential services, 16.3.2020 C(2020) 1753 final; and Communication from the Commission to the European Parliament, the European Council and the Council – Covid19: Temporary Restriction on Non-Essential Travel to the EU, 16.3.2020 COM(2020) 115 final.

51 Communication from the Commission on the implementation of the Green Lanes under the Guidelines for border management measures to protect health and ensure the availability of goods and essential services, 23.3.2020 C(2020) 1897 final.

52 Problems of coordination under the Health Threats Decision were reported already before the crisis; see European Court of Auditors, “Dealing with Serious Cross-Border Threats to Health in the EU: Important Steps Taken but More Needs to Be Done”, special report, 2015; PWC, “Third Independent External Evaluation of the ECDC in Accordance with its Founding Regulation”, 2019.

53 Examples include, among others, export bans of medical equipment, border restrictions and an initial failure to respond to calls for support by Italy.

54 See the overview of measures and timing in Flaxman et al, supra, note 30.

55
ibid.

56 Health Security Committee summary reports from 25 and 30 March 2020, respectively, available at <https://ec.europa.eu/health/hsc_covid19_en>.

57 M Dewatripont, M Goldman, E Muraille and J-P Platteau, “Rapidly Identifying Workers Who Are Immune to COVID-19 and Virus-Free Is a Priority for Restarting the Economy”, *VoxEU.org*, 23 March 2020 <https://voxeu.org/article/rapidly-identifying-workers-who-are-immune-covid-19-and-virus-free-priority-restarting-economy>.

58 J Monras, “Some Thoughts on COVID-19 from a Labour Mobility Perspective: From ‘Red-Zoning’ to ‘Green-Zoning’”, *VoxEU.org*, 25 March 2020 <https://voxeu.org/article/some-thoughts-covid-19-labour-mobility-perspective>.

59 See R Baldwin and B Weder di Mauro (eds), Mitigating the COVID Economic Crisis: Act Fast and Do Whatever It Takes (a VoxEU.org eBook, London, CEPR Press 2020). See also M Draghi, “We Face a War against Coronavirus and Must Mobilise Accordingly”, *Financial Times*, 25 March 2020 <https://www.ft.com/content/c6d2de3a-6ec5-11ea-89df-41bea055720b>.

60 This is where the attention is shifting right now both in the COVID-19 advisory panel and in the Health Security Committee; see Health Security Committee summary reports from 25 and 30 March 2020, supra, note 56; and European Commission’s advisory panel on COVID-19 report from 25 March 2020 <https://ec.europa.eu/health/advisorypanel_covid19_en>.

61 European Commission, “A European Roadmap to Lifting Coronavirus Containment Measures” (Communication 15 April 2020) <https://ec.europa.eu/info/files/european-roadmap-lifting-coronavirus-containment-measures_en>.

62 “Brussels Drops Lockdown Exit Plan after Anger from Capitals”, *Politico*, 7 April 2020 <https://www.politico.eu/article/commission-to-unveil-exit-strategy-as-countries-push-to-lift-corona-measures>.

63 “EU Calls for Co-ordination of Lockdown Exit Strategy”, *Financial Times*, 13 April 2020 <https://www.ft.com/content/ec1ddc00-88dc-4461-b3d6-026ba11b44c7>.

64 See, eg, “Imperial’s Neil Ferguson: ‘We Don’t Have a Clear Exit Strategy’”, *Financial Times*, 7 April 2020 <https://app.ft.com/cms/s/61ed62b5-302f-43f1-b124-cbd614ebffbe.html>.

65 See European Commission, supra, note 61. “[A]t a minimum, Member States should notify each other and the Commission in due time through the Health Security Committee before they announce lifting measures and take into account their views” (p 6). However, “[t]he travel restrictions and border controls currently applied should be lifted once the border regions’ epidemiological situation converges sufficiently and social distancing rules are widely and responsibly applied” (p 12).

66 While the European Commission (supra, note 61) highlights the crucial importance of expanding testing capacity and harmonising testing methodology by Member States, it will act as a mere facilitator in this respect.

